# Analysis of vacuum negative pressure therapy and traditional Chinese medicine lavage in combination with tadalafil for vascular erectile dysfunction

**DOI:** 10.3389/frph.2024.1335239

**Published:** 2024-02-05

**Authors:** Ke Liang, Zunjin Ke, Jianhong Huang, Xiang Fei, Liang Qi, Jie Wang

**Affiliations:** ^1^ Department of Urology, The First People’s Hospital of Pinghu, Pinghu, Zhejiang, China; ^2^ Department of Urology, Zhejiang Hospital, HangZhou, Zhejiang, China

**Keywords:** erectile dysfunction, venous, arterial, negative pressure suction, integration of traditional Chinese and western medicine

## Abstract

This study investigates the clinical effects of the novel Traditional Chinese Medicine (TCM) topical wash used in combination with negative pressure irrigation and tadalafil for the treatment of vascular erectile dysfunction. Eighty-seven patients with vascular erectile dysfunction were divided into an observation group and a control group. The observation group was administered negative pressure irrigation (TCM) in combination with oral tadalafil for four weeks, and the control group was administered oral tadalafil for four weeks. The observation group included 21 patients with arterial erectile dysfunction and 22 with intravenous erectile dysfunction. After treatment, IIEF-5, EHS, GAD scores, PSV, EDV and RI in observation group were improved compared with those before treatment (*P* = 0.000, 0.000, 0.000, L0.000/R0.000, L0.000/R0.000, L0.003/R0.000). Erectile function (IIEF-5, EHS) was significantly improved compared with the control group (*P* = 0.008, 0.002). In the observation group, there were 21 cases of arterial erectile dysfunction and 22 cases of intravenous erectile dysfunction. After treatment, PSV of arterial ED improved significantly (*P* = L0.000/R0.000), but EDV did not decrease significantly (*P* = L0.084/R 0.098). In patients with venous ED, PSV increased (*P* = L0.026/R0.032) and EDV decreased significantly (*P* = L0.000/R0.000). These findings suggest that TCM negative pressure lavage combined with tadalafil improves the blood supply of the penile artery, relaxes smooth muscle, and improves the closing mechanism of venous vessels in patients with vascular erectile dysfunction, ultimately improving the erectile function.

## Introduction

Male erectile dysfunction (ED) is a common disorder that refers to the failure of the penis to achieve or maintain sufficient hardness to meet the needs of sexual life. ED is diagnosed after more than three months of dysfunction (1). Clinically, ED is divided into three types: organic, psychological, or mixed ED (2). Organic ED is the most common type, affecting over 50% of patients with ED (3). The most common organic type of ED is vascular. The first-line treatment for ED is phosphodiesterase 5 inhibitors (PDE5i) (4), which are up to 80% efficacious for ED (5). However, PDE5i are not effective for the treatment of vascular ED, as the underlying vascular lesions and injuries are not treated. In recent years, the efficacies of negative pressure devices and integrated TCM therapies have been validated in clinical practice as research on the mechanisms of vascular ED has increased. In this study, the effects of a new impotence exfoliation prescription for the treatment of vascular ED with negative pressure lavage are investigated.

## Materials and methods

### Ethical approval

Data were collected from 87 patients who underwent prostate puncture biopsy at the Urology Department of The First People's Hospital of Pinghu (Pinghu, China) from December 2022 to August 2023. All patients provided written informed consent. Ethical approval was obtained from The First People's Ethics Committee of Pinghu (approval number 20220726-1).

### Clinical data

The patients were divided into two groups using the random number table method. The observation group included 22 patients with arterial ED [unilateral PSV (Peaksystolicvelocity) <25 cm/s or bilateral PSV sum <50 cm/s] and 21 patients with intravenous ED [EDV(End-diastolic volume) >5 cm/s], with a mean age of 42.49 ± 11.57 years. The mean course of disease was 19.65 ± 17.08 months. The control group included 21 patients with arterial ED and 22 patients with intravenous ED, with a mean age of 40.86 ± 12.46 years. The mean course of disease was 21.14 ± 17.75 months. The groups were similar in terms of IEFF-5(International Index of Erectile Function-5) score, penile hardness scale (EHS) score, anxiety score (GAD), PSA, EDV, and RI(resistance index) (Table 1).

**Table 1:** Comparison of clinical indexes before and after treatment.

Items	Before treatment (*n*)		After treatment (*n*)	
	Observation group (43)	Control group (44)	Observation group (43)	Control group (44)
EHS	1.70 ± 0.56	40.86 ± 12.46	3.07 ± 0.59^*,#^	2.68 ± 0.52
IIEF-5	10.91 ± 3.60	11.05 ± 3.28	19.40 ± 3.19*,^#^	17.70 ± 2.62*
GAD	5.47 ± 1.20	5.73 ± 1.25	4.44 ± 0.93*	4.20 ± 0.88*
PSV (cm/s)				
L	30.60 ± 16.22	28.95 ± 14.69	38.91 ± 14.70*,^#^	33.18 ± 9.26*
R	29.44 ± 14.26	28.41 ± 13.79	37.94 ± 15.18*,^#^	32.39 ± 10.22*
EDV (cm/s)				
L	6.55 ± 4.21	6.21 ± 3.92	4.02 ± 2.02*	4.69 ± 2.44*
R	6.28 ± 3.98	6.07 ± 3.98	3.80 ± 2.05*,^#^	4.9 ± 2.86*
RI				
L	0.88 ± 0.16	0.86 ± 0.17	0.97 ± 0.23*,^#^	0.88 ± 0.21*
R	0.86 ± 0.17	0.85 ± 0.17	1.01 ± 0.19*,^#^	0.90 ± 0.33*

PSV, peak systolic pressure velocity; EDV, end-diastolic velocity; IIEF-5, international erectile function score; L, left; R, right. Compared with before treatment.

* *p* < 0.05. Compared with the control group.

^#^ *p* < 0.05.

### Patient inclusion and exclusion criteria

All patients included in the study were males aged 20–65 years with a permanent sexual partner and an IIEF-5 score of 8–21, which was used to diagnose mild to moderate ED. Vascular ED was diagnosed using penile artery color ultrasound (prior to the examination, 2 ml of alprostil was injected) based on the following parameters: unilateral PSV <25 cm/s or bilateral PSV sum <50 cm/s or EDV >5 cm/s (6, 7). The patients in this study were not administered PDE5i within one month prior to enrollment. All patients had a history of ED longer than six months, with symptoms of blood stasis according to the “Guiding Principles of Clinical Research on the treatment of impotence with new Chinese Medicine” (8). In addition, all patients consented to participate in the study.

Patients with psychological, hormonal, or neurogenic ED were excluded from the study, as were patients with a history of prostatectomy or pelvic surgery. Patients with poorly controlled chronic diseases such as hypertension (systolic blood pressure ≥160 mmHg or diastolic blood pressure ≥100 mmHg) or diabetes (fasting blood glucose >7.0 mmol/L) were also excluded from the study. Patients with abnormal penis or genital structure, penile implants, serious heart disease, cardiac pacemakers, malignant tumors, coagulation dysfunction, or infectious diseases were also excluded from the study.

Patients who could not use the medication or suction under negative pressure as required by the test protocol or undergo treatment, examination, and provision of the relevant research data were released from the study, as were patients who used other treatments during the study period. Patients with adverse reactions to the treatment that were difficult to tolerate and those that were lost to follow-up were also released.

### Treatment plan

The Traditional Chinese Medicine (TCM) prescription of external washing for impotence includes Angelica (15 g), safflower (10 g), honeysuckle vine (20 g), Xianling spleen (15 g), Borneol (3 g), cinnamon (10 g), mint (10 g), clove (10 g), snake seed (15 g), Astragalus (20 g), sapwood (15 g), and chicken spatholobi (20 g). The prescription was prepared at the hospital pharmacy.

**Figure 1:**
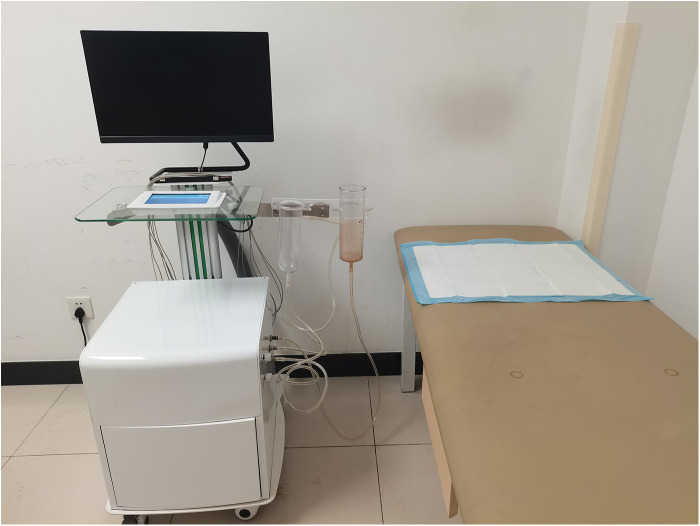
ASW-3501 type men's negative pressure physiotherapy device.

**Figure 2:**
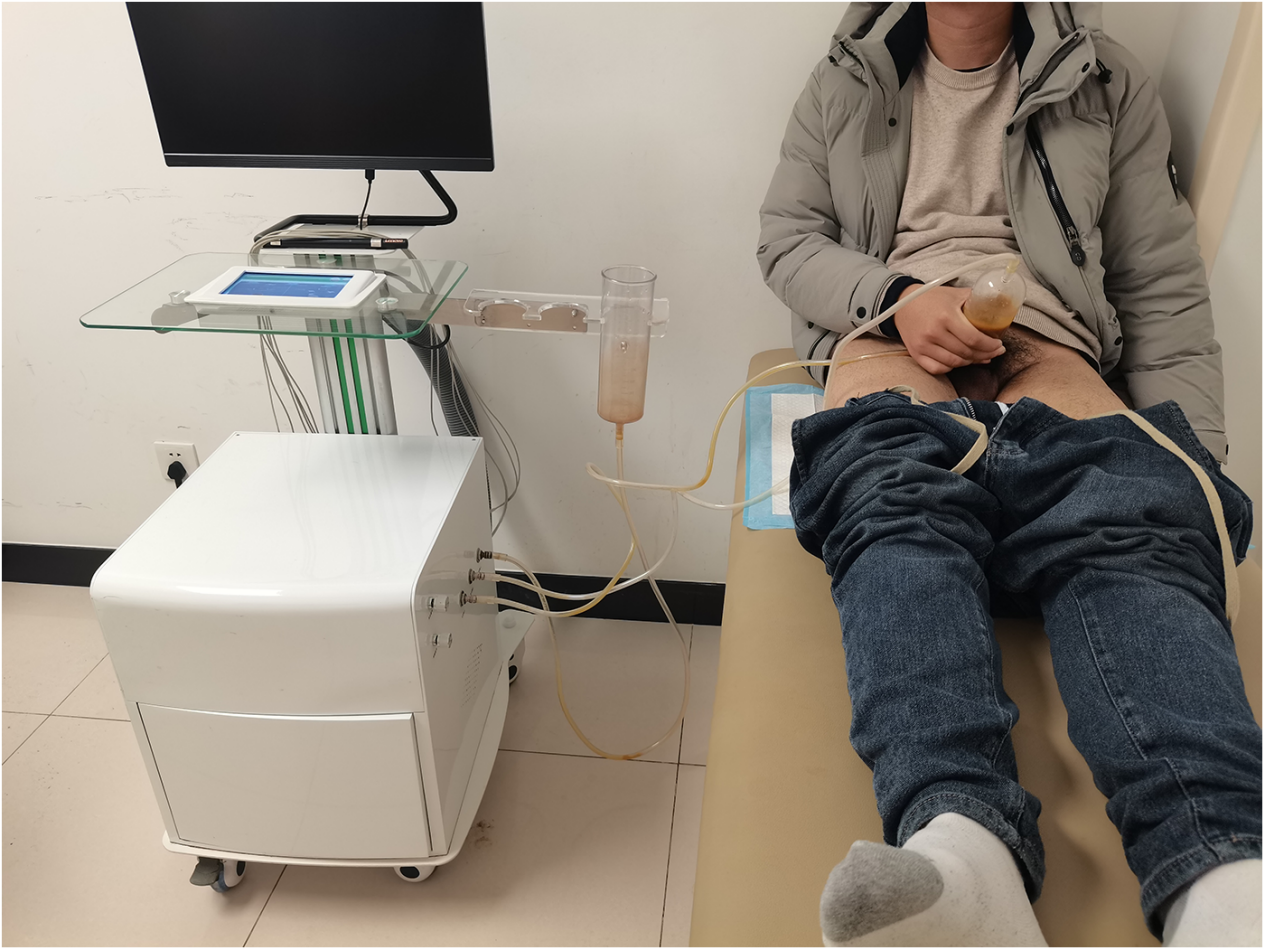
The patient was treated with Chinese medicine negative pressure lavage.

A SW-3501 type men's negative pressure physiotherapy device (3D Medical Technology Jiangsu Co., Ltd., Xuzhou, China) was used, and 250 ml of external washing Chinese medicine was added into the penis sleeve at approximately 37°C. The penis was soaked in the washing at a negative pressure of 0.01–0.03 KPa for 20–30 min. This treatment was repeated eight times every four weeks (Figures 1, 2).

Tadalafil, produced by Dr. Shi from Shandong Luoxin Pharmaceutical Group Co., Ltd. (approval number: H20213052, specification 0.5 mg × 14 tablets) was also prescribed. The patients were administered 0.5 mg tadalafil each night before bed for four weeks.

The observational group underwent TCM irrigation and negative pressure aspiration and was administered tadalafil for four weeks. The control group was administered tadalafil for four weeks.

### Observation Index

The patients' erectile functions were evaluated after the four-week treatment period. The PSV, EDV, RI, and IIEF-5, EHS, and GAD scores were compared before and after treatment.

### Statistical analyses

All analyses were conducted using SPSS version 25.0. The data are expressed as mean and standard deviation. The group *t*-test was used to compare variables between the groups, while the paired sample *t*-test was used to compare variables within the groups before and after treatment. Statistical significance was set at *P* < 0.05.

## Results

### Comparison of IIEF-5, EHS, and GAD scores before and after treatment

After treatment, the IIEF-5, EHS, and GAD scores of the observation group were 19.40 ± 3.19 points, 3.07 ± 0.59 points, and 4.44 ± 0.93 points, respectively, which were statistically different than the scores prior to treatment treatment (*P* = 0.000, 0.000, 0.000). The post-treatment IIEF-5, EHS, and GAD scores of the control group were 17.70 ± 2.62 points, 2.68 ± 0.52 points, and 4.20 ± 0.88 points, respectively, were also significantly different than the pre-treatment scores (*P* = 0.000, 0.000, 0.000). After treatment, the IIEF-5 and EHS scores were better in the observation group than in the control group (*P* = 0.008, 0.002) (Table 1).

### Changes of penile hemodynamics after treatment of arterial ED

The post-treatment PSA levels of patients in the observation group with arterial ED were significantly higher than those before treatment (*P* = L0.000/R0.000). The RI levels were significantly higher after treatment (*P* = L0.025/R0.018). However, the EDV did not significantly change after treatment (Table 2).

**Table 2:** Comparison of penile hemodynamics indexes before treatment of arterial ED.

Group	*N*	PSV		EDV		RI	
		Left	Right	Left	Right	Left	Right
Before treatment	21	18.11 ± 5.76	19.16 ± 3.56	2.81 ± 1.24	2.65 ± 1.16	0.94 ± 0.15	0.93 ± 0.15
After treatment	21	31.8 ± 13.01	32.89 ± 14.64	2.41 ± 1.05	2.27 ± 1.00	1.01 ± 0.14	1.01 ± 0.12
T	–	−4.940	−4.059	1.819	1.738	−2.426	−2.584
P	–	0.000	0.001	0.084	0.098	0.025	0.018

### Changes of penile hemodynamics after treatment of intravenous ED

The bilateral PSA was significantly higher after treatment than before treatment among patients with intravenous ED (*P* = L0.026/R0.032). The bilateral EDV significantly decreased after treatment (*P* = L0.000/R0.000). The RI levels were significantly higher after treatment (*P* = L0.038/R0.001). The EDV did not change significantly after treatment in patients with intravenous ED (Table 3).

**Table 3:** Comparison of penile hemodynamics indexes before intravenous ED treatment.

Group	*N*	PSV		EDV		RI	
		Left	Right	Left	Right	Left	Right
Before treatment	22	42.52 ± 13.77	39.23 ± 13.72	10.10 ± 2.62	9.62 ± 2.17	0.82 ± 0.16	0.78 ± 0.15
After treatment	22	45.70 ± 13.12	42.76 ± 14.39	5.56 ± 1.42	5.25 ± 1.72	0.98 ± 0.33	1.02 ± 0.25
T	–	−2.401	−2.292	0.302	9.634	−2.212	−3.884
P	–	0.026	0.032	0.000	0.000	0.038	0.001

## Discussion

Erectile function requires the coordination and interaction of multiple organ systems, including the endocrine system, blood vessels, and nervous system (9). After sexual stimulation, the erectile center of the hypothalamus releases neurotransmitters to the parasympathetic nerve in the cavernous body of the penis. L-arginine in the non-adrenal non-cholinergic receptor endings and cavernous endothelial cells produce nitric oxide (NO) under the action of nNOS (neuronal nitric oxide synthase) and eNOS (endothelial nitric oxide synthase), stimulating the translation activation enzyme and increasing local cGMP. Consequently, the myosin light chain of spongy smooth muscle cells is dephosphorylated (10), resulting in sufficient dilation of the artery, and compression and closure of the penile sub-albumen vein, preventing blood from flowing out of the penis to maintain an erection (11). When this equilibrium is disturbed, ED occurs.

Vascular ED is classified into arterial and venous types. Arterial ED and cardiovascular and cerebrovascular diseases share the similar risk factors including aging, diabetes, dyslipidemia, hypertension, obesity, metabolic syndrome, sedentary lifestyle, and smoking (2). These factors can induce cavernopathy, including apoptosis and fibrosis of cavernous smooth muscle cells, vascular endothelial dysfunction, and neuropathy and inhibit the release of NO, which affects erectile function. Venous ED is often associated with venous insufficiency caused by valve loss (12). The erection of the penis mainly depends on the blood supply to the cavernous artery, and the PSV is an important indicator of the function of the cavernous artery. EDV is used to determine the closing function of the venous vessels (13). When the cavernous artery is congested, the pressure in the sponge increases, obstructing venous outflow. the internal pressure of the cavernous penis maintains a dynamic balance (6). RI is the most sensitive indicator of perivascular pressure; an RI <0.8 indicates the possibility of an arteriovenous fistula (14).

The use of negative pressure suction devices in the treatment of ED dates back to 1874, when John applied a vacuum negative pressure device made of glass to the penis of a patient with ED to induce erection. Nadig (15) reported the application of negative pressure aspiration in 35 patients with ED, among whom 32 patients had significantly improved symptoms and 30 patients were satisfied with the curative effect. Since then, penile negative pressure suction devices have been widely used in clinics and are constantly undergoing revisions to achieve better efficacy. Zhang et al. (16) confirmed that the vacuum effect of negative pressure suction has anti-fibrosis and anti-oxidation effects and can improve the blood oxygen status of the local corpus cavernosum, resulting in passive dilation of the corpus cavernosum of the penis. Its mechanism of action is primarily to excite the blood vessels and muscles of the cavernous body via negative pressure, resulting in increased NO release. Increased blood flow in the cavernous body helps improve the function of endothelial cells, further promotes the release of NO, activates the NO/cGMP signaling pathway, and maintains the erection (17). In a recent study of 56 middle-aged and elderly patients with ED who underwent vacuum suction therapy, 96% of the patients believed that the device improved their ability to achieve an erection and 94% believed that they resumed satisfactory sexual activity after vacuum suction therapy (13).

The TCM negative pressure suction device used to treat ED is based on the TCM principle that the main lesions of ED are in the liver and kidney channels. The basic pathogenesis is a deficiency of Yin and fluid and excess dryness and heat (18). Vascular ED is similar to blood stasis in TCM; the dysfunction of the vas deferens is minor, resulting in difficulty filling the blood vessels of the penis, and the treatment is mainly aimed at promoting blood stasis. The red flower and cauligodon in Vioxx prescriptions promote blood circulation and channeling, improving blood microcirculation and anti-oxidation. Honeysuckle vine dredges up wind, removes collaterals, and is anti-inflammatory and an antioxidant. Cinnamon has invigorating fire to strengthen the yang, promoting blood circulation and menstruation. Angelica Gan's warmth and moistness can pass through blood stagnation, toning down blood deficiency. Fairy spleen, snake seed, and morinda are warm kidney products that improve the body's metabolism by toning the kidneys and promoting blood circulation. The external washing formula composed of various TCM ingredients used in combination with the negative pressure suction device can lead to dilation of the blood vessels of the cavernous body, improving the endothelial function, promoting blood flow, relaxing smooth muscle, and thickening the white membrane, ultimately promoting the release of NO to maintain a penile erection (19). Previous studies (20, 21) have reported that drugs that promote blood circulation, remove blood stasis, warm the kidney, and assist Yang can protect the function of vascular endothelial cells, providing a theoretical basis for the treatment of ED. Rongzhi et al. (22) reported that TCM lavage combined with negative pressure improves the vascular endothelial function of patients with ED and has a significant effect on the treatment of diabetic ED.

In this study, tadalafil combined with a TCM negative pressure lavage had a significant effect on the treatment of vascular ED, as exhibited by the significantly higher PSV after treatment, leading to an improved blood supply to the cavernous artery, and restoration of the normal erection of the penis. The vasculature of the penile vein is normal in patients with arterial ED; therefore, the reduction in EDV is not significant. In patients with venous ED, the PSV significantly increased and the EDV significantly decreased after treatment, suggesting that the increased blood supply to the penile arteries relaxed the smooth muscles and improved the closing mechanism of the venous vessels, which may not directly affect the venous valves.

In summary, TCM negative pressure lavage can improve the therapeutic effect of tadalafil in patients with vascular ED and is effective against different types of vascular ED. However, the sample size of this study was small, and large-sample studies are required to validate these results.

## References

[1] Male diseases Expert Consensus Group, Beijing Society of Chinese Medicine. Expert consensus on the treatment of erectile dysfunction with integrated medicine of Chinese and western medicine. Chin J Androl. `021) 35(4):59–61.

[2] Domes T Najafabadi BT Roberts M Jeffrey C Ryan F Phil B Canadian urological association guideline erectile dysfunction. Can Urol Assoc J. (2021) 15(10):310–22. 10.5489/cuaj.7572 34665713 PMC8525522

[3] Cui W Li H Guan R Li M Yang B Ch Xu Zh W Efficacy and safety of novel low-intensity pulsed ultrasound (LIPUS) in treating mild to moderate erectile dysfunction: a multicenter, randomized, double-blind, sham-controlled clinical study. Transl Androl Urol. (2019) 8(4):307–19. 10.21037/tau.2019.07.03 31555554 PMC6732092

[4] Ahmed KE Dalia SE Mohammad A El-Gamil Mohammad AH Ashraf H. Advancements in phosphodiesterase 5 inhibitors: unveiling present and future perspectives. Pharmaceuticals (Basel). (2023) 16(9):1266. 10.3390/ph16091266 37765073 PMC10536424

[5] Pyrgidis N Mykoniatis I Haidich AB El-Gamil Mohammad AH Ashraf HA The effect of phosphodiesterase-type 5 inhibitors on erectile function an overview of systematic reviews. Front Pharmacol. (2021) 12:735708. 10.3389/fphar.2021.735708 34557099 PMC8452927

[6] Huang WL Tung SY Tseng CS Wang TD Lee WJ Chen JH The flow index provides a comprehensive assessment of erectile dysfunction by combining blood flow velocity and vascular diameter. Sci Rep. (2022) 12(1):2045–2322. 10.1038/s41598-022-19364-5 36167958 PMC9515177

[7] Corona G Cucinotta D Lorenzo GD Ferlin A Giagulli VA Gnessi L The Italian society of andrology and sexual medicine (SIAMS), along with ten other Italian scientific societies, guidelines on the diagnosis and management of erectile dysfunction. J Endocrinol Invest. (2023) 46(6):1241–74. 10.1007/s40618-023-02015-5 36698034 PMC9876440

[8] Ministry of Health of China. Guiding Principles of Clinical Research on the Treatment of Impotence with New Chinese Medicine. Ministry of Health of China (1993).

[9] MacDonald SM Burnett AL. Physiology of erection and pathophysiology of erectile dysfunction. Urol Clin North Am. (2021) 48(4):513–25. 10.1016/j.ucl.2021.06.009 34602172

[10] Argiolas A Argiolas FM Argiolas G Maria RM. Erectile dysfunction treatments, advances and new therapeutic strategies. Brain Sci. (2023) 13(5):2076–3425. 10.3390/brainsci13050802 PMC10216368 37239274

[11] Gratzke C Angulo J Chitaley K Dai YT Kim NN Paick JS Anatomy, physiology, and pathophysiology of erectile dysfunction. J Sex Med. (2010) 7:445–75. 10.1111/j.1743-6109.2009.01624.x 20092448

[12] Hoppe H Diehm N. Percutaneous treatment of venous erectile dysfunction. Front Cardiovasc Med. (2020) 7:626943. 10.3389/fcvm.2020.626943 33604356 PMC7884342

[13] Beaudreau SA Van Moorleghem K Dodd SM Victoria LJ Christine EG. Satisfaction with a vacuum constriction device for erectile dysfunction among middle-aged and older veterans. Clin Gerontol. (2021) 44(3):307–15. 10.1080/07317115.2020.1823922 33012263

[14] Chen L Xu L Wang J Li H Zhang DQ Zhang CH Diagnostic accuracy of different criteria of pharmaco-penile duplex sonography for venous erectile dysfunction. J Ultrasound Med. (2019) 38(10):2739–48. 10.1002/jum.14982 30839126 PMC6849731

[15] Nadig PW. Six years' experience with the vacuum constriction device. Int J Impot Res. (1989) 1:55–8. 10.1016/S0363-5023(77)80070-1

[16] Zhang DL. Adding a vacuum erection device to regular use of tadalafil improves penile rehabilitation after posterior urethroplasty. Asian J Androl. (2019) 21(6):582–6. 10.4103/aja.aja_34_19 31169141 PMC6859665

[17] Diehm N Pelz S Kalka C Keo HH Mohan V Schumacher MC Do DD Hoppe H. Venous leak embolization in patients with venogenic erectile dysfunction via deep dorsal penile vein access: safety and early efficacy. Cardiovasc Intervent Radiol. (2023) 46(5):610–6. 10.1007/s00270-023-03412-2 36949182 PMC10156837

[18] Zhou H Sun Z Xiang Z. Erdibiejia decoction combined with tadalafil in treating 35 cases of diabetic erectile dysfunction. J Nanjing Univ Chin Med. (2015) 31(5):491–3. 10.14148/j.issn.1672?0482.2015.0491

[19] Tang R-Z Zeng Y Huang X-K Zhong L Huang ZHF Li S. Effect of self-made bushen shengjing decoction combined with negative pressure suction combined with traditional Chinese medicine lavage on serum testosterone level in patients with renal deficiency type diabetes erectile dysfunction. J Changchun Univ Chin Med. (2017) 33(4):589–92. 10.13463/j.cnki.cczyy.2017.04.025

[20] Xu D Zhang Y Bai J Huixing Y Tao W Jihong L Botanical drugs for treating erectile dysfunction: clinical evidence. Front Pharmacol. (2023) 14:1232774. 10.3389/fphar.2023.1232774 37654605 PMC10467024

[21] Xu R-N Ma J-X Zhang X Liao Z-D Fu Y-J Lv B-D Efficacy of Chinese herbal medicine formula in the treatment of mild to moderate erectile dysfunction: study protocol for a multi-center, randomized, double-blinded, placebo-controlled clinical trial. Int J Gen Med. (2023) 16:5501–13. 10.2147/IJGM.S436347 38034900 PMC10683653

[22] Cheng HS Zhang T Zhang SS Mo XW Li HS. A randomized controlled clinical trial of tongluo zhanshi decoction in the treatment of male erectile dysfunction (kidney deficiency and blood stasis type). Chin Pharmacovigil. (2015) 12(5):264–266.270. CNKI:SUN:YWJJ.0.2015-05-003

